# Calidad de la atención en podología universitaria. Análisis del cuestionario de satisfacción del paciente

**DOI:** 10.1016/j.aprim.2025.103297

**Published:** 2025-07-16

**Authors:** Manuel Yván Arnao Villegas

**Affiliations:** Universidad Tecnológica del Perú, Lima, Perú

*Sr. Editor,*

Es de suma importancia destacar la relevancia del rol que desempeñan los podólogos en la prevención, diagnóstico y tratamiento de diversas afecciones relacionadas con los pies, puesto que su labor no solo se centra en tratar problemas comunes de los pies, sino también en la detección temprana de enfermedades graves como la diabetes, trastornos vasculares y artritis, lo cual puede afectar significativamente la salud podológica y, en consecuencia, la calidad de vida de las personas^1^.

El presente estudio aborda la calidad de atención en podología a través de un cuestionario y elabora un análisis de satisfacción del paciente. Es importante mejorar la calidad del apoyo asistencial a los pacientes que ofrece la Clínica Universitaria de Podología de la Universidad de A Coruña, donde se llevó a cabo un estudio con el objetivo de evaluar la validez y fiabilidad de un cuestionario de satisfacción del paciente y analizar la percepción de la atención, en el cual colaboraron en este entorno docente-asistencial con la participación de 315 usuarios adultos, teniendo como resultado una puntuación media global de 4,48 ± 0,34 (buena)^2^.

El análisis factorial del estudio reveló 6 dimensiones en lugar de las 5 originales, lo que evidencia una estructura más válida. La consistencia interna fue alta (alfa de Cronbach de 0,881). Los factores mejor valorados fueron «Trato», «Trámites» y «Aspectos clínicos». Los hallazgos indican que existe una alta satisfacción con la atención al paciente en la Clínica Universitaria de Podología de la Universidad de A Coruña^2^.

Por otro lado, los cuestionarios de satisfacción constituyen una herramienta fundamental para medir y mejorar la calidad del servicio en la podología universitaria. De una forma más eficiente y cercana al paciente, se pueden obtener resultados favorables^3^. Por ello, la calidad asistencial no solo depende del dominio técnico, sino también de la capacidad de los profesionales para comprender y atender con empatía las necesidades de los pacientes^4^.

En conclusión, el uso del cuestionario de satisfacción del paciente evidencia que la calidad de la atención en podología universitaria de la Universidad de A Coruña puede ser evaluada de manera confiable y válida a través de este instrumento, porque mejora así la percepción de los pacientes sobre diversos aspectos del servicio, permitiendo identificar fortalezas y áreas de mejora en la práctica clínica. Consideramos que los podólogos son profesionales esenciales, cuyo trabajo no solo mejora la calidad de vida de los pacientes, sino que también optimiza la eficiencia del sistema de salud.

## Comité ético

Por la naturaleza del trabajo, no fue necesaria una evaluación del comité ético. Tampoco se precisó consentimiento, pues no se evaluaron pacientes para la Carta al Editor.

## Financiación

Este trabajo no ha recibido ningún tipo de financiación.

## Conflicto de intereses

El autor declara no tener ningún conflicto de intereses.
